# Infected Hydrocele of the Canal of Nuck

**DOI:** 10.1155/2013/275257

**Published:** 2013-12-04

**Authors:** Parkash Mandhan, Zaid Raouf, Khalid Bhatti

**Affiliations:** ^1^Division of Paediatric Surgery, Department of Surgery, Sultan Qaboos University Hospital, 123 Muscat, Oman; ^2^Department of Surgery, Hamad General Hospital, Hamad Medical Corporation, P.O. Box 3050, Doha, Qatar; ^3^Department of Surgery, Sultan Qaboos University Hospital, 123 Muscat, Oman

## Abstract

Hydrocele of the canal of Nuck in children is rare. It may present as incarcerated inguinal hernia and necessitates emergency exploration. Risk of infection in hydrocele of the canal of nuck is very rare. We present a case report of a 5-year-old girl who presented with a left tender inguinolabial region swelling with fever, tachycardia, and mild dehydration. The clinical features were suggestive of strangulated left inguinal hernia and further imaging and surgical exploration revealed it to be an infected hydrocele of the canal of Nuck. High ligation and hydrocelectomy were performed. Hydrocele of the canal of Nuck in a female child presenting with an inguinal swelling should be considered in differential diagnosis.

## 1. Introduction

Contents of the inguinal canal vary between female and male population. During development, in the female fetus, round ligament of the uterus descends into the inguinal canal to the labium major. The peritoneal fold that descends with the round ligament is named the canal of Nuck. If this communication fails to close, it results in an indirect hernia or a hydrocele [1].

Hydrocele of the canal of Nuck is not a common entity as only isolated case reports are published in the literature [1–6]. Infection is an uncommon complication of hydroceles in children, and until now only 5 cases have been reported in the English language literature, all in boys [2, 7].

We present a case of infected hydrocele of the canal of Nuck in a 5-year-old girl, initially pictured as strangulated inguinal hernia and later diagnosed as a case of infected hydrocele of the canal of Nuck. We emphasize to consider this as a differential diagnosis in girls with tender inguinolabial swelling.

## 2. Case Report

**Figure 1:**
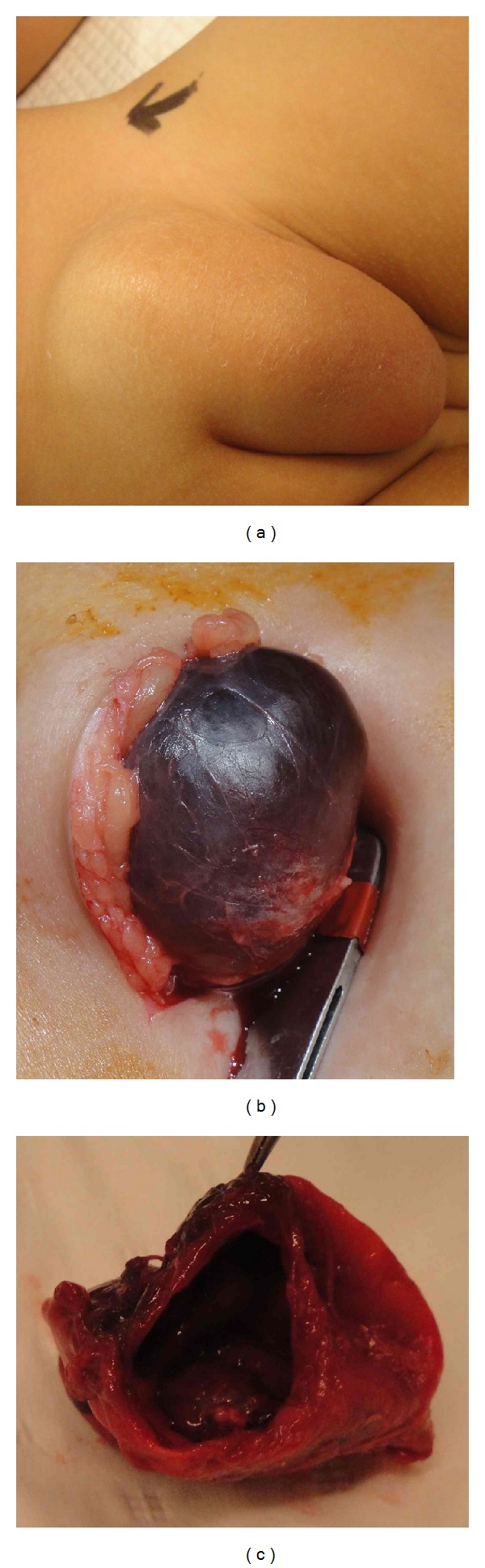
Operative images of left inguinolabial region showing (a) swelling extending down to ipsilateral labium majus, (b) tense encysted cystic swelling containing dark-brown fluid, and (c) excised and opened hydrocele.

A 5-year-old girl presented to the emergency with history of painful mass in the left inguinolabial region noticed a week before presentation. The child has a positive history of trivial trauma in school while playing with other children. The inguinal swelling and pain got gradually worse over the week. There was no history of recent illness in near future. Examination revealed temperature of 38°C, heart rate of 140/m, and mild dehydration. Abdominal and chest examinations were unremarkable. Local examination revealed a 6 × 3 cm irreducible tense swelling in the left inguinolabial region, with overlying skin redness and edematous. Blood investigations revealed white blood cell count of 14000/mm^3^, C-reactive protein 50, normal electrolytes, and coagulation profile. Her urinalysis was normal. Blood culture and sensitivity was sent and was reported normal. An ultrasound of inguinal region performed in the emergency room revealed a cystic mass in left inguinal region containing fluid in the upper part and fluid with internal echoes in the lower part of mass, and there was no free fluid noted in the abdomen. After resuscitation with fluids and administration of IV antibiotics, she was taken to the operating room for exploration. Operative findings included a cystic mass in the left inguinal canal extending down to the ipsilateral labia majora (Figure 1(a)), cyst containing dark brown fluid in the proximal part, and clotted blood with dark fluid in the distal part (Figure 1(b)). The cystic swelling was confirmed to be an encysted hydrocele of canal of the Nuck with no evidence of associated inguinal hernia. The cyst was easily isolated from the surrounding edematous tissue planes and after complete separation high ligation of the canal of Nuck was performed and cyst was excised (Figure 1(c)). Postoperative course was uneventful. Microbiology of fluid collected from cyst was negative and cytology showed foamy macrophages and scattered lymphocytes and neutrophils. Histopathology of excised cyst revealed inflammatory cells infiltrated in the hydrocele sac with some degenerate blood and cholesterol clefts. No epithelial lining was seen and calretinin staining showed a few mesothelial cells. The histology findings were consistent with infected hydrocele of the canal of Nuck. In followup at 6 weeks, 6 months and 18 months after surgery, patient is well and there is no swelling or recurrence on the operated side.

## 3. Discussion

In the female child, hydrocele of the canal of Nuck usually presents as a painless, translucent, fluctuating, and nonreducible swelling in the inguinal area and labium majora. Counseller and Black classified hydrocele of canal of Nuck into 3 types [8]. The most common type, which corresponds to encysted hydrocele of the cord in male, is one with no communication with peritoneal cavity forming an encysted fluid collection along the tract of descent, from the inguinal ring to the vulva. The second type corresponds to communicating hydrocele in male when there is a persistent communication with the peritoneal cavity. A third type is a combination of the two as a result of the inguinal ring constricting the hydrocele like a belt so that part is communicating and part is enclosed, giving this the name of hour glass type. In our case, the findings were corresponding with type I encysted hydrocele.

The infection in the hydrocele of the canal of Nuck in female children is not known and has been reported only in boys where it has been considered to happen due to seeding from intraperitoneal source through patent processus [2, 7]. In our patient, this hypothesis of intraperitoneal seeding is not applicable as there were no peritoneal signs on initial abdominal examination and also ultrasound was negative for any intra-abdominal fluid. However, in our view the initial trauma she had in school may have been directly responsible for minor bleeding inside the processus vaginalis, later this small hematoma got infected and caused associated symptoms, pain and tenderness on the affected inguinolabial side mimicking for strangulated inguinal henna or inguinal abscess.

Hydrocele of the canal of Nuck should be considered in differential diagnosis for inguinal swelling in a girl. Clinically this may mimic indirect inguinal hernia, femoral hernia, abscess, tender adenopathy, Bartholin's cyst, posttraumatic hematoma, and rare entities such as cystic lymphangioma, neuroblastoma metastasis in groin, and ganglion [1, 9, 10]. Further evaluation with ultrasound and MRI has been utilized to reach correct diagnosis [6]. In our case, clinical findings and ultrasound facilitated the prompt diagnosis and we proceed with surgical exploration without any further delay.

In conclusion, a hydrocele of the canal of Nuck though rare should be considered in the differential diagnosis in female children presenting with an inguinal swelling. Ascertaining a definitive diagnosis on physical examination may not be possible; hence, further evaluation with ultrasound imaging will be helpful. Surgery is mandatory for final diagnosis and treatment.
